# Professor Delong Su: a pioneer in Schistosomiasis Eradication in China

**DOI:** 10.1093/procel/pwac048

**Published:** 2023-01-06

**Authors:** Zhimei Que, Zhujun Su, Yuanyuan Meng

**Affiliations:** Family of Professor Delong Su, Shanghai 200031, China; Family of Professor Delong Su, Shanghai 200031, China; Beijing Institutes of Life Science, Chinese Academy of Sciences, Beijing, China

Delong Su (Teh-long SU, 1906–1985) was an internationally renowned public health expert, a medical educator and philosopher, and one of the major founders of classic epidemiology in China (Fig. 1). He was among the first group of the national distinguished professors and doctoral supervisors appointed since the founding of the People’s Republic of China. Dr. Delong Su served as vice-president of the Shanghai First Medical College and vice-chairman of the National Schistosomiasis Research Council. He was elected as an honorary member by the International Society of Epidemiology, the only Chinese epidemiologist among 44 world-renowned epidemiologists. He was the first Chinese expert nominated for Léon Bernard Foundation Prize, a very prestigious award for these with accomplished outstanding service in the field of social medicine by the World Health Organization (WHO) because he was the world’s pioneer who fully elucidated the distribution of snails and ecology of schistosomiasis and applied his research in the elimination of schistosomiasis in China. Professor Su created an ecological method to eradicate snails and screened a series of measures on eliminating snails and preventing schistosomiasis infection. His economical innovation named “anti-cercaria coat” and “anti-cercaria pen” were awarded the National Science Award. Dr. Delong Su edited the first national textbook of *Epidemiology* (three times as chief editor), which laid the foundation for epidemiology research in China, and founded the Society of Epidemiology, Chinese Medical Association, serving as the first President of the Society of Epidemiology. Professor Delong Su made great contributions to public health in China and the world, such as preventing and controlling a variety of major epidemics, including Schistosomiasis, a plague ravaging China for more than two thousand years, liver cancer, and cholera. Especially, his outstanding achievements in Schistosomiasis control have made a significant and brilliant chapter in the history of public health in China.

**Figure 1.**
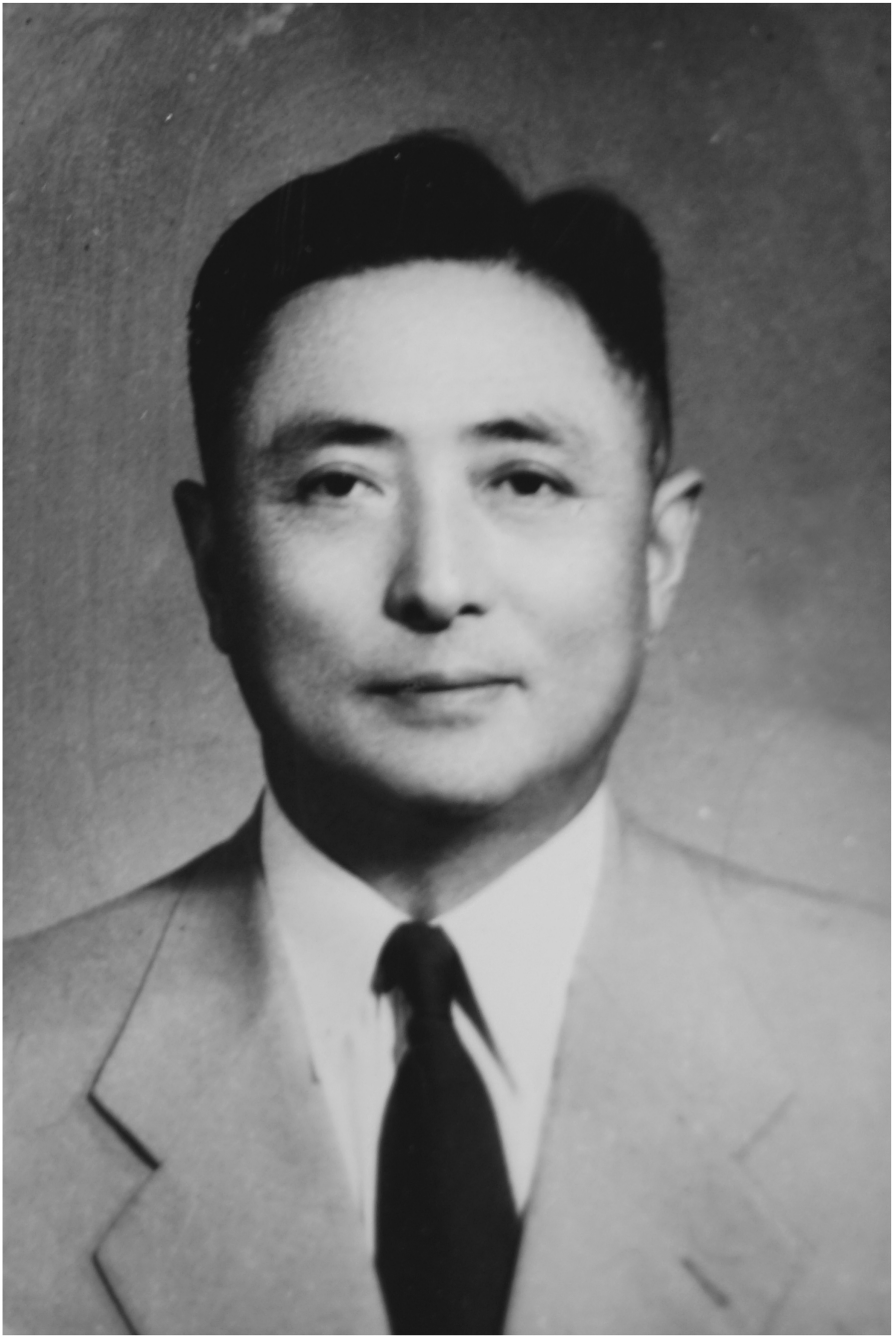
Professor Delong Su (Teh-long SU) (1906–1985).

## Encountering the “pestilence,” falling in love with the public health

In 1906, Delong Su was born in a Christian family in Nanjing, the ancient capital of the six dynasties. His parents lived on small handicrafts and his family was very poor. Delong Su was the eldest of the five surviving children in the family. He was intelligent from an early age, and began to learn English in the third grade of primary school by himself. With excellent academic performance, Delong Su graduated from a Christian middle school with a scholarship. For the next 2 years, he dropped out of school because of poverty, but still tried his best to study by himself. Although Delong Su had received an English education for a long time, he had an excellent foundation in the Chinese language, and his heart was to become a leader who adhered to the core of four Chinese words “Xiu Qi Zhi Ping,” meaning self-cultivation, family harmony, national governance, and world peace.

In 1927, Delong Su was first admitted to the pre-medical program of the National Central University in Nanjing and then admitted to the National Shanghai Medical College. When he was in college, he joined the *Natural Science Society of China* and participated in the founding of *Science World* magazine, published popular science articles, and enthusiastically popularized medical knowledge for public education. In the summer of 1931, over a thousand people suffered from acute schistosomiasis after swimming in Qingyang Port, Kunshan county, a suburb of Shanghai. At that time, the medical community knew little about the disease and it was difficult to diagnose. Delong Su presented a report at the school’s journal club, elaborating the damage of schistosomiasis, and considering it as a disease worthwhile to study. This was the first time he paid attention to an infectious disease, which was harmful to human beings.

**Figure 2.**
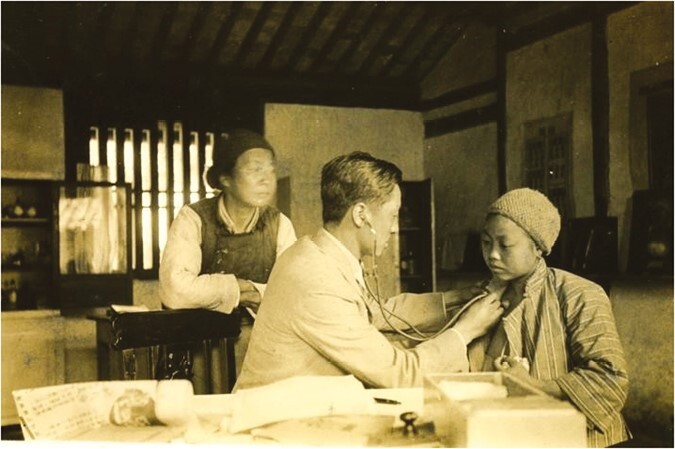
Delong Su (middle) offered a free clinic for farmers in 1935.

In 1935, Delong Su received his M.D. degree and successfully graduated as a top student among his cohort. He received a Gold Medal and CUM LAUDE certificate because of his academic excellence. Dr. Su stayed in the same school as an Assistant Professor. He established a “Rural Health Center” in Zhuanqiao town, Shanghai where he started a new health education center of the National Shanghai Medical College, and conducted epidemiological investigations (Fig. 2). This process gave him extensive experience in preventing and treating patients with schistosomiasis.

## Studying overseas, exploring new knowledge

In October 1944, Dr. Delong Su was awarded a scholarship from the Rockefeller Foundation of the USA and continued his study as a Master’s student at the School of Public Health, Johns Hopkins University in the USA. He was mentored by well-known Professors, Drs. Kenneth F. Maxcy and Lowell Jacob Reed. His study at Johns Hopkins University helped him learn epidemiological methods and establish the concept of mathematical modeling in epidemiology, which made a solid foundation for his further epidemiologic study and public health education. In 1945, Dr. Delong Su graduated from Johns Hopkins University with a Master’s degree in Public Health (MPH).

**Figure 3.**
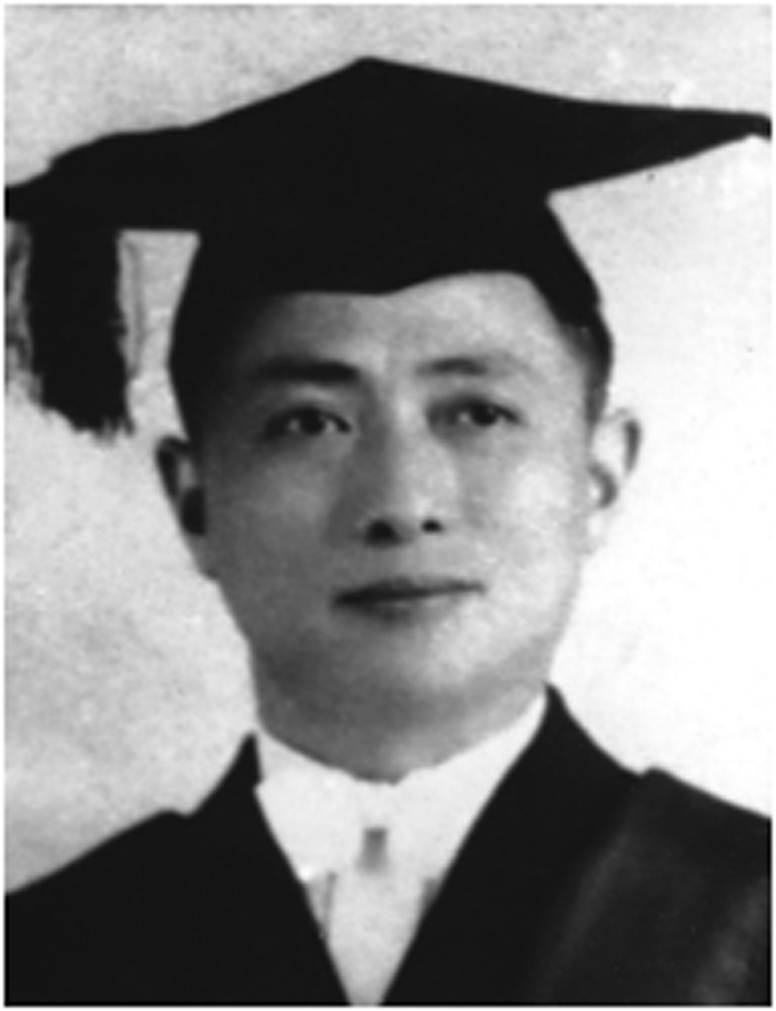
Delong Su received his DPhil from the Oxford University in 1947.

In the fall of 1945, Dr. Delong Su went to Oxford University to further pursue his doctoral studies (Fig. 3). His doctoral advisor, Professor Howard Walter Florey was one of the inventors of penicillin and a Nobel laureate. During his stay at Oxford University, Dr. Delong Su mastered basic experimental skills in chemistry, microbiology, and pathology, and gained a solid basis on experimental medicine. He also studied medical statistics from Professor John Alfred Ryle, the chair of the Institute of Social Medicine at the University of Oxford. In 1947, Dr. Delong Su first-ever discovered and isolated a powerful antibiotic from sewage in the world, and named it *Micrococcin*. Reuters reported this innovative discovery to the world as a piece of scientific news. In the same year, Dr. Delong Su received a Doctor of Philosophy (DPhil) and was selected as member of the Royal Statistical Society and Microbiological Society, which were extremely rare among Chinese at that time. Dr. Su participated in the First General Assembly of the World Medical Association in Paris in September 1947 representing the Chinese Medical Association. On June 24, 1948, he attended the First World Health Assembly of the United Nations convened in Geneva, Switzerland. At the end of 1948, Dr. Delong Su declined the enthusiastic retention of his mentor, Professor Florey, and returned to China. He brought back scientific research instruments and reagents purchased by his personal savings. He was appointed as a professor of epidemiology at National Shanghai Medical College, his alma mater, and as the chair of the departments of public health and microbiology. For over 40 years, Dr. Su rebuilt the department of public health of the National Shanghai Medical College from a very small department with only a few people into a well known and influential school of public health in the world, created a group of public health disciplines with Chinese characteristics, and trained a large number of experts and leaders in medicine, medical education and public health, as well as health administrators for China. Dr. Delong Su had made outstanding contributions to the research and education for the development of public health and medicine.

## “Schistosomiasis must be eliminated”

Schistosomiasis has been prevalent in China for at least 2000 years and was known as the “god of plague” that ravaged 13 provinces, cities, and regions in southern China including important agricultural provinces and economic areas in China. In the early days after the People’s Republic of China was established in 1949, ~100 million people were affected, and more than 13 million people suffered from schistosomiasis. Throughout the 1940s, almost no articles about schistosomiasis were published by Chinese scholars. Dr. Delong Su reviewed the western literature after World War II and published a long review article, entitled “*The Progress of Schistosoma japonicum Research in recent years*” in *Chinese Medical Journal* in 1950. This article and the need to improve Chinese people’s health played an important role in the process of changing his academic research direction on Chinese schistosomiasis research, prevention and control, and embarking on a long journey to eliminate schistosomiasis.

Snails were recognized as the “only intermediate host” of Schistosoma. Eradicating snails was a key preventive measure to control schistosomiasis at that time, while there were no proven measures and drugs to eliminate snails, because most of these measures stayed in the hypothetical and laboratory experimental stage. Dr. Delong Su was determined to change the situation by using the vast countryside fields as his nature laboratory. He worked with his students, conducted a variety of experiments, made observations in the experimental and education fields, and explored more economical and effective measures to eliminate snails. According to the suggestion of Dr. Su, people in most of the area in the village carried out extensive health education activities and campaigns of eliminating eggs of Schistosoma by storage-manure, reinforcing the management of feces, changing the consumption of river water to well water, and shutting off potential routes of infection with polluted water among villagers. These preventive measures had been considered the most important tasks in the prevention of schistosomiasis at that time.

In 1953, Dr. Delong Su initiated snail ecology research in China. In 1954, he proved in his experiments that ammonia produced from the decomposition of urinary urea in human urine can kill the eggs of Schistosoma. This cost-free method promoted the practice of feces bio-safety disposal, and became an effective way to block the transmission of schistosomiasis in the water-network region in the south of the Yangtze River. Dr. Delong Su observed the responses of snails in relation to changes of lights and temperatures through laboratory and on-the-spot fields. It was determined that the temperature of 13°C and the light intensity of 3600 Lux at dawn and dusk were the most suitable for snails, making it easier to identify them. He also studied the changing rule of snails in different seasons, providing a scientific basis for regional characteristics of snail distribution areas in China. In the earlier days, people put a lot of manpower and resources into eliminating snails above the river waterline, but gained fruitlessly. Dr. Su insisted on an evidence-based prevention strategy by translating research results into preventive practice. He overturned the opinion in textbooks that snails had hibernating habits. After persistent research for 1 year, Dr. Su found the distribution regularity of snails above and under the river waterline, which was significant for mastering the living condition of snails and even eliminating snails. According to his suggestion, the water-network region in the south of the Yangtze River had completely changed the strategy for snail control and elimination.

At the same time, Dr. Delong Su started to test chemical methods for snail elimination. From chemical screening to laboratory experiments, and eventually on-site experiments, Dr. Su participated in all these steps in person. Dr. Delong Su would have never stopped his research experiments if he were still alive. He pioneered the “paper money method” to screen molluscicides, and this multi-purpose, efficient, and inexpensive method won the praise from colleagues worldwide. In more than 20 years, he screened a variety of molluscicidal drugs, such as calcium arsenate, calcium arsenite, tea seed cake, and so on. Later, he successively discovered that ethylenediamine and urea were effective to snails and nontoxic to fish. More important, urea used for snail control in fish ponds was not only safe and harmless to fish, but also can fatten fish.

**Figure 4.**
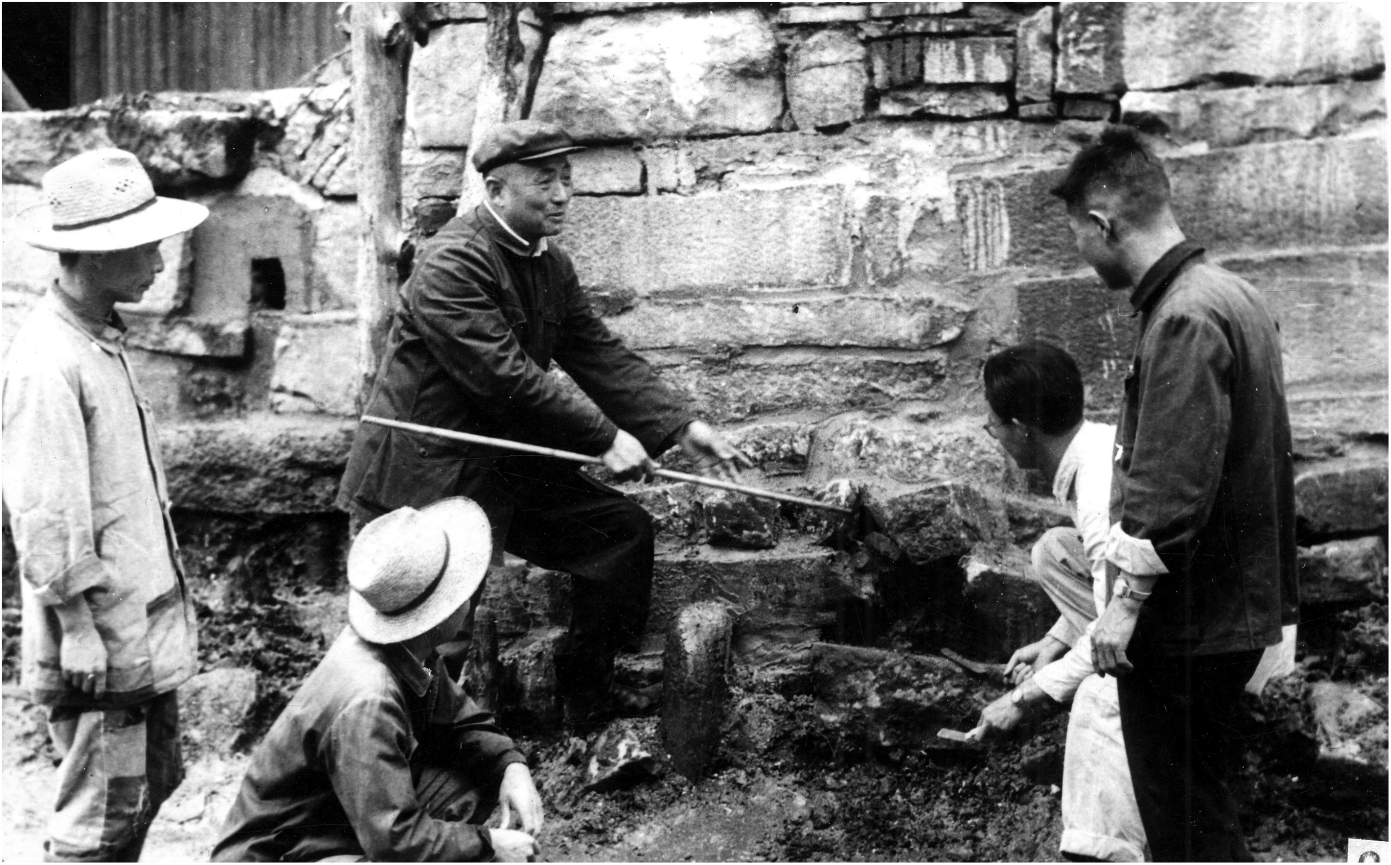
Delong Su (middle) instructed schistosomiasis control and snail elimination in Qingpu county in 1965.

Since 1950, Delong Su had conducted research every year in Qingpu county, one of the top ten schistosomiasis endemic areas in China (Fig. 4). In 1958, when QingPu county was incorporated into Shanghai city, 40% of the population were infected with schistosomiasis. The distribution area of patients and snails was similar to that of the endemic areas in Japan. Professor Delong Su proposed to establish an experimental field with a research focus in QingPu county, aiming to carry out control against schistosomiasis. Leading more than 20 young teachers and technicians together with the local people and farmers, snails were eliminated in villages and towns one by one. In this process, he employed chemical methods that achieved great effects. In 1963, Dr. Delong Su’s paper “Negative Binomial Distribution of Snails” was published, which was the first research achievement in the world to comprehensively explain the distribution patterns of snails.

In the early 1970s, although facing adversity, Dr. Su still continued his research and completed a project of niclosamide against cercariae of schistosomes. According to that, Su invented the “anti-cercaria coat” and “anti-cercaria pen.” The efficacy of “anti-cercaria coat” remained unchanged after tens of thousands of times after mud grinding and water flushing. “Anti-cercaria coat” can still prevent the invasion of schistosome cercariae after it was hung in the open air for several months or preserved indoors for more than 20 years. The “anti-cercaria pen” owned a remarkable effect, although it only cost a few RMB cents. After rubbing the skin with mud for 8 h, the “anti-cercaria pen” still had a protective effect. The solution can be used as a skin protective agent and can also prevent rice field dermatitis. It had no discomfort on the skin and did not pollute clothes. This invention was very popular with farmers. In 1975, his inventions passed technical appraisal and were promoted and brought to Africa. In the late 1970s, the WHO, the Admiralty of the USA, and the University of Cambridge in the UK all sent letters to ask for samples, starving for the understanding of formulations and transferring the related technology. The two inventions were awarded the National Conference on Medical Science Prize and Shanghai Major Award for Scientific Research in 1978.

In August 1981, Professor Delong Su delivered a speech, entitled “Ecological Study of Schistosomiasis in China,” at the 9th Scientific Conference of the International Society of Epidemiology in Edinburgh, Scotland. The scientific achievements of schistosomiasis control in China, which were based on prevention and control, had been praised by international academic experts. Dr. Delong Su was the first scholar who fully elucidated the ecology of schistosomiasis in China, and his publication, *Ecology of Schistosomiasis in China*, indicated the directions of schistosomiasis control. He dialectically analyzed the potential impact on schistosomiasis with the construction of the Three Gorges of the Yangtze River, which was enjoyed at a high degree of evaluation at home and abroad. In January 1982, Dr. Delong Su was nominated for Léon Bernard Foundation Prize by WHO, who was the first Chinese to be nominated for this award.

In November 1985, Shanghai announced the elimination of schistosomiasis completely. In recognition of Dr. Delong Su’s great contributions to schistosomiasis prevention and control, he was posthumously awarded the distinguished pioneer and researcher on schistosomiasis control and scored great meritorious service. On 9 November 2018, the Chinese Preventive Medicine Association posthumously awarded Professor Delong Su the title of “National Pioneer of Schistosomiasis Control.”

**Figure 5.**
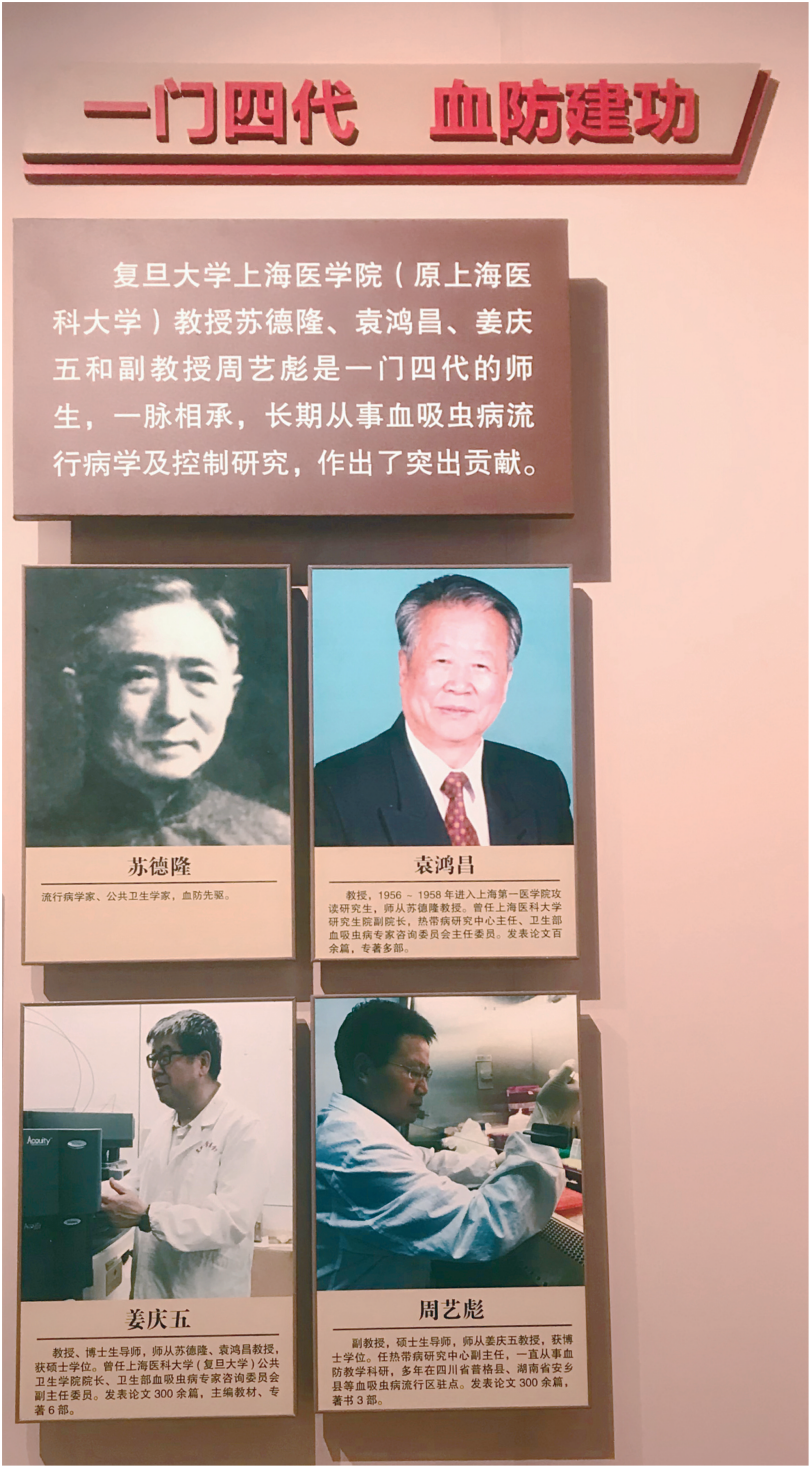
Exhibition board in China Schistosomiasis Memorial Hall.

In China Schistosomiasis Prevention Museum, a special exhibition board, entitled “Achievements by four generations, Merits of schistosomiasis prevent,” had been set up to record the outstanding contributions and unique status made by the professors and students of Shanghai Medical College headed by Professor Delong Su for schistosomiasis prevention in China for over 70 years (Fig. 5). Under the efforts made by generations of researchers in schistosomiasis control led by Dr. Delong Su, the schistosomiasis control program in China had gained great achievements and made China become a great example in the world. Most of the scientific achievements in schistosomiasis control in China had been shared in the world. Chinese prevention products and standards have been applied to other schistosomiasis endemic countries and made a great contribution to the global control of schistosomiasis.

“The needs of my country were my research goal,” Dr. Delong Su said. His lifetime exploring and pursuing truth in his research and practice was still widely recognized by the scientific community. His great personality of tolerance was remembered by his colleagues and future generations. His idea of giving a high priority to prevention and transforming the environment to eliminate diseases was deeply rooted in the hearts of public health researchers. His scientific spirit also inspired generations of intellectuals to work hard for people’s health and to build world-class medical colleges with Chinese characteristics and the world’s top universities!
